# Long lasting insecticidal net use and its associated factors in Limmu Seka District, South West Ethiopia

**DOI:** 10.1186/s12889-018-5022-8

**Published:** 2018-01-10

**Authors:** Mitiku Teshome Hambisa, Tessema Debela, Yadeta Dessie, Tesfaye Gobena

**Affiliations:** 1 School of Public Health, Haramaya University College of Health and Medical Sciences, P. O. Box 235, Harar, Ethiopia; 2 Research Centre for Generational Health and Ageing, University of Newcastle, P.O. Box 2308, Callaghan, Newcastle, NSW Australia; 3 Oromia Regional Health Bureau (ORHB), Public Health Emergency Management (PHEM) and Health Research Team, Addis Ababa, Ethiopia; 4 Department of Environmental Health Sciences, Haramaya University College of Health and Medical Sciences, P. O. Box 235, Harar, Ethiopia

**Keywords:** LLIN, Malaria, Ethiopia

## Abstract

**Background:**

Many countries in sub-Saharan Africa, including Ethiopia, are focusing on the distribution of Long Lasting Insecticidal Nets (LLINs) to combat malaria. However, utilization of the LLIN is low when compared with LLIN possession because of various factors. This study was conducted to measure the actual LLIN usage and identify factors associated with its utilization in Limmu Seka District, South West Ethiopia.

**Methods:**

A community based cross-sectional survey was conducted among 830 households from December 25, 2011 to February 29, 2012.

**Results:**

A total of 830 households were selected by stratified systematic sampling and surveyed. Ninety percent of those surveyed owned LLINs and 68.3% reported that someone had slept under the net on the night prior to the survey. The factors associated with LLIN usage were knowledge of the mode of malaria transmission (AOR; 0.086, 95% CI 0.03, 0.24), the preferred conical shapes of the LLIN (AOR; 1.6, 95% CI 1.31, 4.1), receiving information about their use from Health Extension Workers (HEWs) (AOR; 2.4, 95% CI 1.5, 3.9), hearing media campaigns (AOR; 3.2 95% CI 3.5, 9.2), education at a health facility (AOR; 2 95% CI 1.5, 3.9) or having a family size of three or less (AOR; 2.1, 95% CI 1.3, 3.5).

**Conclusion:**

Although ownership of Long Lasting Insecticidal Nets was high at 90%, the actual usage of LLIN was low, and not all family members were protected. Promoting the usage of LLINs utilization by those at most risk, especially the conical shaped ones, through intensified health education using HEWs and mass media campaigns at all health facilities, schools and communities will improve LLIN utilization.

## Background

The World Health Organization (WHO) has reported that half of the world’s populations was at risk of malaria at the start of 2016 and in 2015 alone, there were 212 million malaria cases and 429,000 deaths worldwide [1]. Populations living in sub-Saharan Africa still carry the highest malaria burden according to the 2016 World Malaria Report. About 90% of cases and 92% of all malaria deaths are estimated to occur in Africa [1]. Children under 5 years of age (86%) and pregnant women were most severely affected [1]. In addition, 125 million pregnant women are exposed to malaria each year of which more than 30 million live in the African region [2]. Consequently, nearly 10,000 women and 200,000 children die annually in African region alone [3].

Malaria is one of the leading causes of morbidity and mortality in Ethiopia. According to the Ethiopian Federal Ministry of Health’s (FMOH)-‘Health and Health Related Indicators’ [4], malaria is second among the top 10 causes of morbidity, the third cause for hospital admission, and the third cause for mortality. Over three million cases of malaria (both confirmed and clinically diagnosed) were reported in Ethiopia in 2012 [4]. It is the major public health problem in terms of morbidity and burden on health care facilities, accounting for an increasing percentage of outpatient consultations in most health facilities in the Oromia region [5].

There is strong evidence that LLINs can provide a substantial degree of protection against mortality and morbidity from malaria especially when used by a high proportion of the population. This can be achieved through LLIN use causing a reduction in either or both of the larvae and adult mosquitoes [6]. Among malaria control and prevention activities, the use of LLINs can reduce up to 90% of malaria transmission [7].

Ethiopia launched a control program to scale up efforts and relieve the malaria burden in the third quarter of 2005 [6]. The FMOH identified four major areas of intervention for malaria control which includes: disease management, selective vector control, epidemic prevention and control, prevention and control of malaria in pregnancy [6]. Long Lasting Insecticidal Nets ownership will have little impact on the burden of malaria unless people sleep under the net and many large-scale programs have encountered challenges in the consistent use of LLINs [8]. Although an increasing number of studies have documented LLINs ownership, only a few studies have systematically investigated the actual usage of LLINs which has a greater influence on malaria morbidity trends [9]. Consistent use of LLIN can reduce malaria mortality by 20% and morbidity by 50% in children less than 5 years of age. When combined with early diagnosis and treatment, it can reduce malaria mortality by over 50% [10]. However, there is little documented research as to the factors that promote LLIN usage, as some of the previous studies were not focused on the utilization of the net. Ensuring healthy lives and well-being for all is a key Sustainable Development Goal (SDG), thus the findings from this study can provide an insight into malaria control for health policy and programs, and guide further research. The purpose of this study was to assess LLIN use and its associated factors in Limmu Seka District, South West Ethiopia.

## Methods

### Study setting

The study was conducted in Limmu Seka District, Jimma Zone, South Western Ethiopia from December 25, 2011 to February 29, 2012. The study design was a community based cross-sectional study. We used a single population proportion formula to determine the sample size. It was computed by taking the proportion of households who were utilizing their LLINs (56.4%) as documented in the annual reports of the Jimma Zone Health Department in the year 2010 [11] and considering a 95% confidence level, 5% margin of error, design effect of 2 and 10% non- response rate. The final sample size was 830 households. From 38 kebeles (the smallest administrative region in Ethiopia) in the district, 12 kebeles were selected randomly by stratified sampling and 830 households (study participants) were selected by a systematic random sampling method after proportional allocation of the sample to each kebele.

### Data collection

Data were collected using structured questionnaires adapted from the literature [5, 12–14] and the 2007 Malaria Indicators Survey (MIS) questionnaires used in Ethiopia. Data collection tools were pre-tested on 30 households. Questionnaires were translated into the local language, Afan Oromo.

### Variables

The main outcome variable for the study was the usage of LLINs. Independent variables included, knowledge-related factors (the perceived cause of malaria, its transmissions, prevention and misconceptions about LLINs use), socio-demographic factors (educational status of the head of the household, family size, occupation, sex, and religion), LLINs characteristics (net’s age, shape and colour preference), and inter-household factors (conditions of the house, number of rooms, family size).

### Data analysis

We analyzed the data using the Statistical Package for Social Science (SPSS) Version 16 for Windows. Odds ratio and 95% confidence intervals were used to determine the associations between independent variables and the outcome variable. Multivariate logistic regression analysis was used to assess the effect of independent variables on the dependent variable.

### Ethical considerations

We obtained a signed written consent from the participants before commencement of the interview. Ethical approval was obtained from Haramaya University, College of Health and Medical Sciences, Institutional Health Research Review Ethics Committee (IHRREC). The data were only accessed by investigators, and records were kept confidential.

## Results

### Socio-demographic characteristics

A total of 830 households were surveyed resulting in a response rate of 100%. Of 830 households included in the study, 74% (614) of the respondents were heads of the household and the remaining were other household members. The median age of the respondent was 32 years. Family size ranged from 1 to 14, with a mean of 5.63 persons per household. The surveyed households had a total of 4673 family members of which 10.7% (498) were children under 5 years and 3.8% (177) were pregnant women. Most of the respondents (78.4%) were farmers by occupation and almost half of them had not attended formal education (Table 1).

**Table 1:** Socio-demographic characteristics of the respondents on LLINs utilization in Limmu Seka District, South West Ethiopia, April, 2012

Variables	Frequency (*n* = 830)	Percentage
Sex of respondent		
Male	512	61.7
Female	318	38.3
Religion of respondent		
Muslim	740	89.1
Orthodox	66	8
Protestant	24	2.9
Occupation of respondents		
Farmer	651	78.4
Merchant	79	9.5
House wife	48	5.8
Governmental employer	46	5.5
Others	7	0.7
Ethnicity of the respondent		
Oromo	753	90.7
Amhara	31	3.7
Keficho	18	2.2
Gurage	17	2
Others (Dawuro, Yems)	11	1.4
Educational status of the respondent		
Illiterate	411	49.5
Read and write	166	20
Grade 1–4	99	11.9
Grade 5–8	86	10.4
Grade9–10	28	3.4
Grade 11–12	14	1.7
Grade 12+	26	3.1

### Malaria related knowledge and perception

Almost all of the participants (99.8%) reported they had heard of malaria as a health problem. About 67 % (553) of the participants answered that mosquito bites were the cause of malaria while other common answers included close contact with a malaria patient, eating or drinking with a patient and cold weather. Similarly, 91.2% of the study households recognized fever as a symptom of malaria, while the rest of the respondents associated malaria with body pains, headache, loss of appetite, chills and shivering, nausea and vomiting. Two-thirds (67%) of the respondents believed that malaria can cause death, whereas the rest did not have such beliefs. The majority of participants (89.6%) believed that malaria is a preventable disease. The most commonly mentioned preventive strategies by the respondents were eliminating mosquito breeding sites 76.9% (572) and impregnated bed nets use 92.3% (686).

### Possession and utilization of bed net

**Fig. 1:**
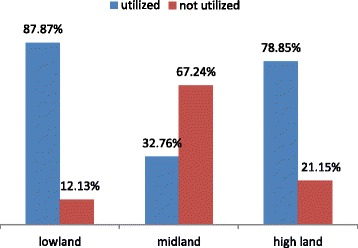
LLINs utilization according to land topography in Limmu Seka District, Southwest Ethiopia, April, 2012

The average number of bed nets per household was 0.9 while the average household family size was 5.63. Approximately 90% (745) of households owned at least one LLIN. Almost all had complete knowledge about the proper use of LLINs. Out of the 745 households that owned LLINs, 68.3% (509) reported that someone had slept under the LLIN the prior night. Children under 5 years of age had greater utilization of LLINs than pregnant mothers (59.2% vs 52.5%, *P* = 0.1218). Only half of the study participants reported using LLINs throughout the year (Table 2). There was a wide geographical variation in the utilization of LLINs. A higher proportions of lowlanders were utilizing the net as compared with the highland and midland populations (Fig. 1).

**Table 2:** Long Lasting Insecticidal Nets possession and utilization in Limmu Seka District, South Western Ethiopia, April, 2012

Variables	Frequency (*n* = 830)	Percentage
LLINs		
possession		
Yes	745	89.8
No	85	10.2
utilization		
Yes	509	68.3
No	236	31.7
Who uses the bed net		
Under fives (*n* = 498)	295	59.2
Pregnant (*n* = 177)	93	52.5
All family (*n* = 4673)	506	12.6
Frequency of LLINs use (*n* = 745)		
Always	274	53.8
Some times	32	6.3
Seasonal	193	37.9
Occasional	10	2
Seasonal use of LLINs (*n* = 745)		
Rainy season	37	7.3
After rainy season	35	6.9
Dry season	164	32.2
All over the year	273	53.6
Reason for not using LLINs (*n* = 236)		
causes heat, discomfort	82	30.5
Lack of information	154	65.5

*NB* Multiple responses are possible (for malaria preventive measures)

### Factors associated with bed net utilization

At first different independent variables were considered for bivariate analysis. Finally, we took variables which were significant at *p* ≤ 0.05 to be the final model. In the multivariate analysis, knowledge about the mode of malaria transmission, the conical shape preference of LLINs (conical), small family size, residing in a household with indoor residual spraying (IRS), having information from the mass media, HEWs and/or health facilities were strongly associated with LLINs utilization. Households who did not know the modes of malaria transmission were 91% less likely to utilize nets when compared to those who knew about malaria transmission through mosquito bite (AOR; 0.086, 95% CI 0.03, 0.24). However, households that had received information about the nets from HEWs, the media or a health facility were 2.6, 3.2 and 2 times more likely to have used a net respectively when compared to non-utilizing households (Table 2). Similarly, respondents residing in sprayed houses (AOR; 2.4, 95% CI 1.5, 3.9), who had a family size of three or less (AOR; 2.1, 95% CI 1.3, 3.5) and preferred conical shaped LLINs (conical) (AOR; 1.6, 95% CI 1.31, 4.1) were significantly associated with LLIN usage (Table 3).

**Table 3:** Multivariate analysis showing factors associated with LLINs utilization among households in Limmu Seka District, South West Ethiopia, April, 2012

Variables	LLINs utilization		95%CI	
	Yes (%)	No (%)	COR (95%CI)	AOR (95%CI)
Educational status				
Illiterate	149 (38)	243 (62)	0.20 (0.38, 0.73)	0.86 (0.57, 1.29)
Literate	266 (75.4)	87 (24.6)	1.0	1.0
Information sources				
Media				
No	49 (16.8)	242 (83.2)	1.0	1.0
Yes	218 (53.4)	190 (46.6)	5.7 (3.8, 8.3)	3.2 (3.5, 9.2)*
HEWs				
No	66 (42.9)	88 (57.1)	1.0	1.0
Yes	118 (22.6)	403 (77.4)	2.6 (1.8, 3.7)	2.4 (1.5, 3.9)*
Health facility				
No	425 (71.4)	170 (28.6)	1.0	1.0
Yes	65 (82.3)	14 (17.7)	1.9 (1.01, 3.4)	2 (1.6, 3.8)*
Malaria transmission				
Mosquito bite	147 (23.2)	486 (76.8)	1.0	1.0
Others modes	26 (78.8)	7 (21.2)	0.08 (0.04, 0.19)	0.086 (0.03, 0.24)*
Residing in sprayed household				
No	163 (59.7)	110 (40.3)	1.0	1.0
Yes	330 (85.7)	55 (14.3)	4 (2.8, 5.8)	2.4 (1.5, 3.9)*
Shape preference				
Rectangular	120 (61.2)	76 (38.8)	1.0	1.0
Conical	242 (80.7)	58 (19.3)	2.45 (1.6, 3.8)	1.6 (1.31, 4.1)*
Family size				
≤ 3	269 (71.7)	106 (28.3)	1.4 (1.1, 1.9)	2.1 (1.3, 3.5)*
> 3	240 (64.9)	130 (35.1)	1.0	1.0
Children Presence in household				
No	173 (62.9)	102 (37.1)	1.0	1.0
Yes	336 (71.5)	134 (28.5)	1.5 (1.07, 2.03)	1.07 (0.71, 1.64)

* Statistically significant at *p*-value < 0.05

## Discussion

In this large scale study of 830 households, Long Lasting Insecticidal Net utilization was associated with an understanding of mode of malaria transmission, conical shaped preference of LLINs, lower family size, residing in sprayed houses, and information sourced from the mass media, HEWs or health facilities. The study has revealed 90% of households owned at least one bed net, but only 68.3% of them used their LLIN on the night before the survey. Another study conducted in three regional states of Ethiopia reported similar findings [12]. The assumption that increasing ownership or accessibility will lead to automatic LLIN utilization is incorrect in many cases [12]*.* Previous studies in a rural district of Ethiopia and in selected urban and rural areas of Oromia and Amhara regions have also revealed that not all nets owned by the households were being utilized properly [13]*.* On the other hand, LLIN utilization in this study was higher than a study conducted in Kersa, Eastern Ethiopia in which 33.5% of the households used at least one LLIN the night before the survey [14]. This might be because the majority of the population used the LLIN for other purposes such as wearing it as a headband, undershirt and different types of clothing possibly due to low socio-economic status [8]. Study participants who do not know how malaria is transmitted were not likely utilize nets when compared to those who know about malaria transmission through mosquito bite. Similarly, a study conducted in Wonago District, Southern Ethiopia found the knowledge that LLINs prevent mosquito bites was significantly associated with utilization of LLINs by households and under-five children [5]. This suggests that for proper utilization it is important to know the mode of malaria transmission [5].

The shape of LLINs had a considerable influence on utilization. In the present study, the conical shaped LLIN was strongly associated with LLIN usage. In a similar way, in a study conducted in Oromia and Amhara Regional States of Ethiopia, conical LLINs were more likely to have been used the previous night compared with rectangular LLINs (AOR; 2.27, 95% CI 1.10–4.68) [15]. Previous studies analyzed from Ethiopia and Kenya also reflect this finding [16, 17]. On the other hand, in a study conducted in the Eastern part of the country the blue colour and cylindrical shape LLINs were more preferable than the white and rectangular ones [18]. In our case, the preference might be due to the fact that conical LLINs use a single point for hanging which seems comfortable to use.

There are still a lot of misconceptions regarding knowledge about causes and transmission of malaria in this community. For instance, in this study the role of mosquitoes in malaria transmission was recognized only by 67% of respondents which was lower than the study report in nine malaria prone districts of Ethiopia in which 81% recognized malaria transmission by mosquitoes [19]. Low educational level, external factors of the households participating in health education, less exposure to education that Health Extension Workers provide and less access to health information may be possible reasons [9].

The National Malaria Control and Prevention Strategy recommends the use of LLINs all year round but with high emphasis on peak transmission season [20], while in this study only half of the study participants were using LLINs all year round. This is comparable with the findings from Tigray in which 44% used their LLINs during rainy season, 48.7% after the rainy season and 18.2% all year round [17]. Lack of formal education and information may be a possible explanation. This study has also revealed low levels of LLIN use in pregnant mothers (50.8%) when compared with the under five-year age group (78.1%). This is in contrast with a study conducted in Kenya where mothers used LLINs three times more than children (30% of the mothers and 11% of the children used LLIN). The scenario here is different than that in Kenyan as pregnant women as pregnant women in Kenya tend to receive nets through routine antenatal care (ANC) services [21]*.*

In our study, households who were informed by Health Extension Workers (HEWs) about LLINs were 2.6 times more likely to sleep under a net (AOR; 2.6, 95% CI 1.8, 3.7) when compared to non-user households. Ethiopia has strengthened the health system since 2005 with the establishment and expansion of the Health Extension Program (HEP), allowing closer monitoring of epidemic-precipitating factors at the local level. In this regard, peripheral health services such as HEWs, are vital in preventive and control measures as well as for early detection of malaria epidemics [20]. This finding also indicates that HEWs fulfill important preventive role: they know the socio-cultural background of the community, they teach in a language that the community can understand, they focus on behavioral change communication and they are located in health posts in rural. Families who were informed about the LLINs by a health facility were two times more likely to have used a bed net compared with non-user households (Table 3). Similarly, a study conducted in Nigeria found that the availability of a health facility was a predictor of LLIN utilization [22]. This might be due to the health facilities provided morning health education on the top ten diseases for their patients and clients. It is also known that health facilities are responsible for LLIN distribution and teaching the community about its utilization through HEWs.

Households for whom the media was their source of information about LLINs were also 3.2 times more likely to utilize a net when compared with non-utilizers (Table 3). A similar study assessing the impact of a mass media campaign on bed net use in Cameroon provided strong evidence for the role of mass media communication in encouraging net use [23]. This result also supports mass media communication interventions in support of malaria control strategies such as LLINs [23].

Households having a family size of three or less (AOR; 2.1, 95% CI 1.3, 3.5) were significantly associated with LLIN utilization (Table 3). This might be due to the fact that there is no separate bedroom in rural Ethiopia and the majority of the households owned one LLIN (the average number of bed nets per household in this study was 0.9 while the average household family size was 5.63) so in a family of three, a couple and young child can sleep on one bed. This is supported by a study conducted elsewhere in the country in which the availability of a separate bedroom and possession of two or more LLIN significantly increased LLIN utilization by households and under-five children [5]. In addition, a study conducted in Eastern Nigeria showed that, parents with a family size of less than four used LLIN more than those who had large families [24].

Residing in sprayed houses (AOR; 2.4 95% CI 1.5, 3.9) was strongly associated with LLINs utilization. In the same way, a study conducted in 17 sub-Saharan African countries showed that, living in households with both LLINs and IRS (indoor residual spraying) was associated with a significant reduction of malaria morbidity and mortality [25]. This could be due to the community’s exposure to different malaria prevention and control strategies including LLIN and IRS so that they have had more education. Some other variables for non-use of LLIN such as net age, religion and ethnicity that were found as significant in other studies were not found to be associated with LLIN utilization in this study.

The study had some limitations: There may be recall bias, as people may not accurately remember who slept under the net the previous night and there was no other way of checking. LLIN use in the community is also prone to social desirability bias as the community tends to report socially acceptable behavior and deny socially stigmatized behavior.

## Conclusion

This study found that Long Lasting Insecticidal Net utilization was low compared with its possession. Its utilization was associated with the knowledge of modes of malaria transmission, conical shape preference of LLINs, lower family size, residing in a sprayed household, and having information about LLIN from mass media, HEWs or health facilities. Promoting LLINs utilization, especially the more convenient conical shaped one, through intensified health education and mass media campaigns at all health facilities, schools and communities will improve LLIN utilization.

## Abbreviations

ANC: Antenatal care
AOR: Adjusted Odds ratio
FMOH: Federal Ministry of Health of Ethiopia
HEP: Health Extension Program
HEWs: Health Extension Workers
IHRREC: Institutional Health Research Review Ethics Committee
LLINs: Long lasting insecticidal nets
OR: Odds ratio
WHO: World Health Organization
